# Crystal structure and Hirshfeld surface analysis of 7,7-dimethyl-2-phenyl-3,3a,4,6,7,8,9,9a-octa­hydro-1*H*-benzo[*f*]iso­indole-1,5(2*H*)-dione

**DOI:** 10.1107/S2056989022002353

**Published:** 2022-03-10

**Authors:** Dong Cheng, Xinlei Gao, Jiating Huang, Xiang-Zhen Meng

**Affiliations:** a Department of Chemical and Material Engineering, Chaohu College, Chaohu, People’s Republic of China

**Keywords:** crystal structure, tricyclic oxoisochromene derivatives, Hirshfeld surface analysis, cascade reactions, C—H⋯ inter­actions

## Abstract

The title compound, C_20_H_23_NO_2_, was obtained *via* the reaction of *N*-allyl-*N*-phenyl­acryl­amide with 3-iodo­cyclo­hex-2-en-1-one using PdCl_2_(PPh_3_)_2_ as a catalyst. The compound crystallizes in the monoclinic space group *P*2_1_/*c*. The fused-ring system is not planar and the five- and six-membered rings are *trans*-fused. The mol­ecular geometry is partially stabilized by an intra­molecular C—H⋯O hydrogen bond, forming an *S*(6) ring motif. In the crystal, mol­ecules are linked by C—H⋯O and C—H⋯π inter­actions into a three-dimensional network. To further analyse the inter­molecular inter­actions, a Hirshfeld surface analysis was performed. The results indicate that the most important contributions to the overall surface are from H⋯H (65.5%), O⋯H/H⋯O (17.5%) and C⋯H/H⋯C (14.3%) inter­actions.

## Abstract 2

The crystal structure and Hirshfeld surface analysis of 7,7-dimethyl-2-phenyl-3,3a,4,6,7,8,9,9a-octa­hydro-1*H*-benzo[*f*]iso­indole-1,5(2*H*)-dione obtained *via* the reaction of *N*-allyl-*N*-phenyl­acryl­amide with 3-iodo­cyclo­hex-2-en-1-one using PdCl_2_(PPh_3_)_2_ as a catalyst,, is reported.

## Chemical context

A cascade reaction is a chemical process that comprises at least two consecutive reactions such that each subsequent reaction occurs only by virtue of the chemical functionality formed in the previous step (Nicolaou *et al.*, 2010 ▸; Jash *et al.*, 2019 ▸; Knowles *et al.*, 2021 ▸). Although cascade reactions have been successfully employed for the synthesis of the core skeleton of many important natural products, the design and performance of cascade reactions remain a challenging aspect of organic chemistry (Zhang *et al.*, 2022 ▸; Xie & She, 2021 ▸). Meanwhile, alkyl­ation of the α position of enones and their derivatives has have drawn considerable attention (Krafft *et al.*, 2005 ▸; Muimhneacháin *et al.*, 2017 ▸; Shen & Huang, 2008 ▸; Zhang *et al.*, 2010 ▸; Jana *et al.*, 2021 ▸). McGlacken described a Pd-catalysed coupling procedure for tricyclic oxoisochromene derivatives, which represents an example of the α aryl­ation of activated carbocyclic enone-based substrates (Muimhneacháin *et al.*, 2017 ▸). Huang and co-workers have realized a series of reactions including Sonogashira coupling, propargyl-allenyl isomerization, and [4 + 2] cyclo­addition combined *via* α alkyl­ation of carbocyclic enone-based substrates, affording an efficient and stereoselective synthesis of polycyclic skeletons (Shen & Huang, 2008 ▸). Given this background, we report herein the synthesis and crystal structure of the title compound. 
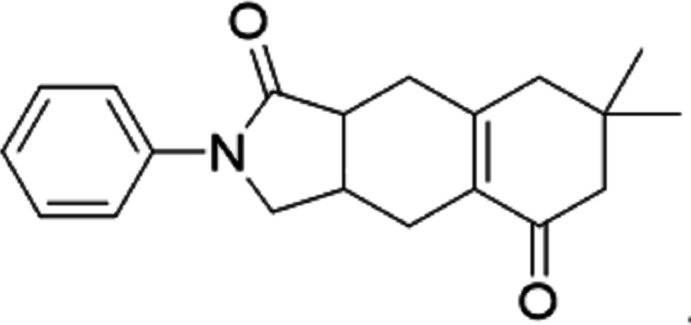


## Structural commentary

**Figure 1:**
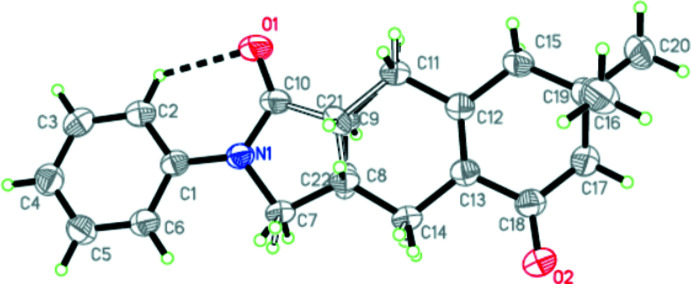
The mol­ecular structure of the title compound, with the atom labelling and displacement ellipsoids drawn at the 50% probability level. H atoms are shown as small circles of arbitrary radii.

The title compound crystallizes in the monoclinic crystal system in space group *P*2_1_/*c*. Its mol­ecular structure is shown in Fig. 1 ▸. The structure of a racemic compound possesses a disordered enanti­omer layout (Jacques *et al.*, 1994 ▸) and atoms C8 and C9 are found to be disordered. They were both split into two fragments (C8/C22 and C9/C21) and were refined. This refinement led to a 0.805 (10): 0.195 (10) occupancy ratio over two positions for C8 and C9. The site occupancies of C8, C9 and C21, C22 are 0.805 (10) and 0.195 (10), respectively. The fused ring system is not planar. The *sp* ^2^-hybridized character of atoms C12 and C13 is confirmed by the C12—C13 [1.350 (3) Å] bond length, and the C11—C12—C15 [114.9 (2)°] and C14—C13—C18 [116.3 (2)°] bond angles. There is a strong intra­molecular hydrogen bond (C2—H2⋯O1; Table 1 ▸), forming an *S*(6) ring motif.

## Supra­molecular features

**Figure 2:**
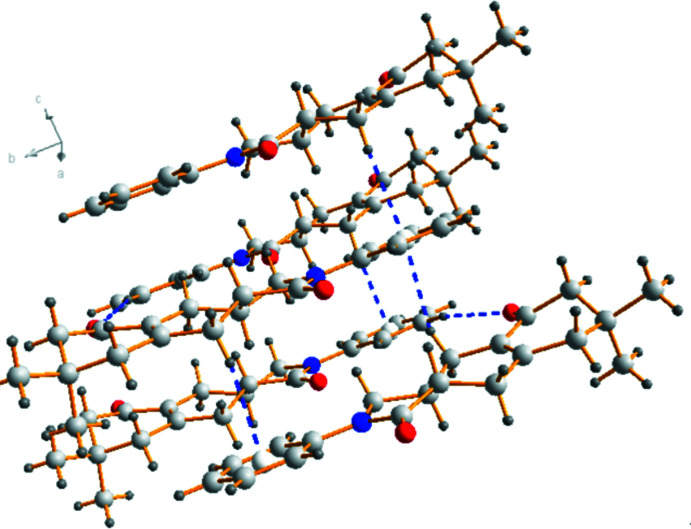
A packing diagram of the title compound. The C—H⋯ π and C—H⋯O inter­actions are shown as dashed lines.

**Figure 3:**
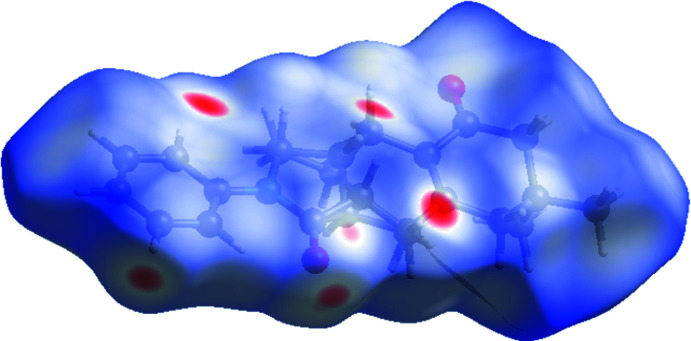
The Hirshfeld surface mapped over *d*_norm_ in the range −0.2740 (red) to 1.7368 (blue) a.u.

**Figure 4:**
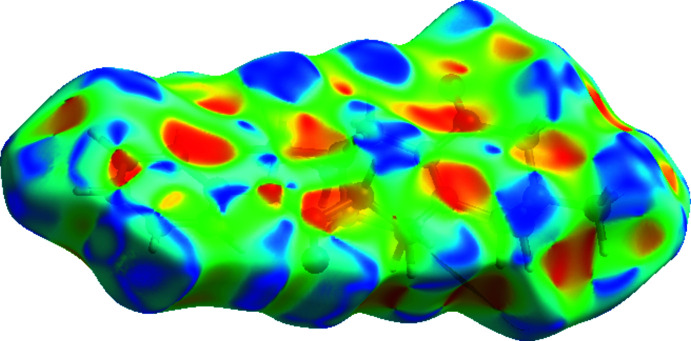
The Hirshfeld surface mapped over shape-index.

**Figure 5:**
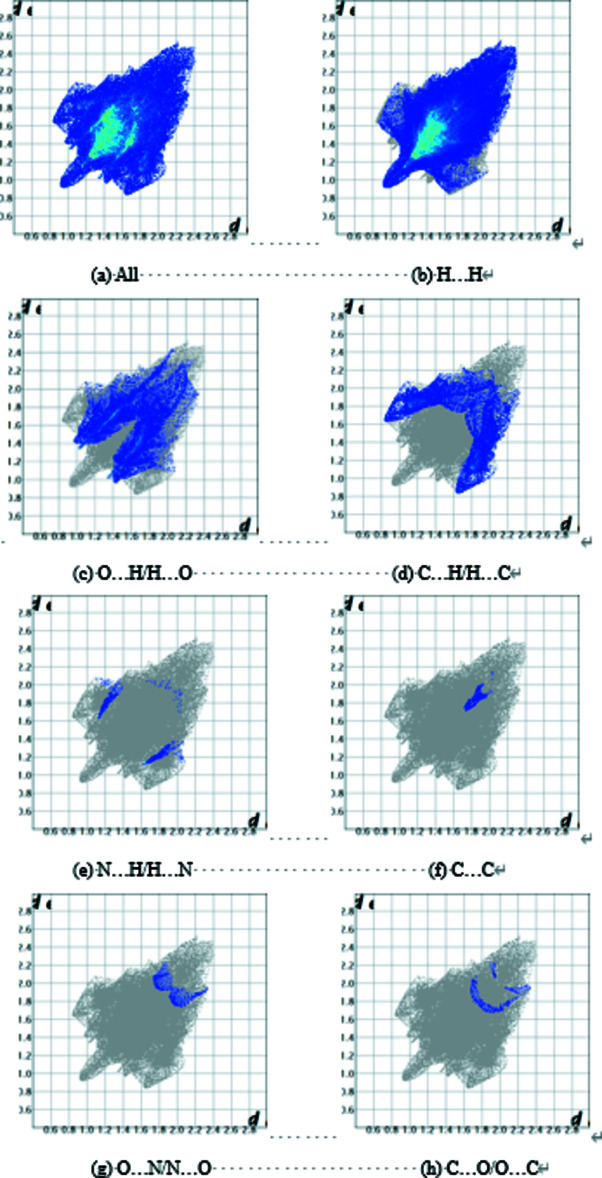
Two-dimensional fingerprint plots for the title compound: (*a*) all inter­molecular inter­actions, (*b*) H⋯H contacts, (*c*) O⋯H/H⋯O contacts, (*d*) C⋯H/H⋯C contacts, (*e*) N⋯H/H⋯N contacts, (*f*) C⋯C contacts, (*g*) O⋯N/N⋯O contacts, (*h*) C⋯O/O⋯C contacts.

The crystal packing of the title compound (Fig. 2 ▸) features inter­molecular C—H⋯O hydrogen bonds and C—H⋯π inter­actions (C3—H3⋯O2^i^; C11—H11⋯*Cg*3^ii^ and C14—H14*A*⋯*Cg*3^iii^ or C11—H11*D*⋯*Cg*3^ii^ and C14—H14*D*⋯*Cg*3^iii^; symmetry codes are given in Table 1 ▸). In the crystal, mol­ecules are stacked together layer by layer. Mol­ecules in same layer are linked by C3—H3⋯O2 inter­actions, forming a layer parallel to the *ab* plane (Fig. 2 ▸); Mol­ecules in different layers are linked by C11—H11*A*⋯*Cg*3 and C14—H14*A*⋯*Cg*3 or C11—H11*D*⋯*Cg*3 and C14—H14*D*⋯*Cg*3 inter­actions (Fig. 2 ▸). In order to investigate the inter­molecular inter­actions in a visual manner, a Hirshfeld surface analysis was performed using *CrystalExplorer* (Spackman & Jayatilaka, 2009 ▸; Turner *et al.*, 2017 ▸). The bright-red spots on the Hirshfeld surface mapped over *d*_norm_ (Fig. 3 ▸) indicate the presence of C—H⋯π and C—H⋯O inter­actions. The absence of adjacent red and blue triangles on the shape-index map (Fig. 4 ▸) suggests that there are no notable π–π inter­actions. The fingerprint plots (Fig. 5 ▸) are given for all contacts, and those delineated into C⋯O/O⋯C (0.4%), O⋯N/N⋯O (0.5%), C⋯C (0.7%), N⋯H/ H⋯N (1.0%), C⋯H/H⋯C (14.3%), H⋯O/O⋯H (17.5%) and H⋯H (65.5%). The most important contributions to the crystal packing are H⋯H and O⋯H/H⋯O contacts.

## Database survey

A search of the Cambridge Structural Database (Version 2021.1; Groom *et al.*, 2016 ▸) for compounds having a 3,3a,4,6,7,8,9,9a-octa­hydro-1*H*-benzo[*f*]iso­indole-1,5(2*H*)-di­one fragment gave two hits, including 2a,8,9b-trimethyl-3,4,6,6a,9a,9b-hexa­hydro­[2]benzofuro[1,7-*ef*]iso­indole-2,5,7,9(2a*H*,8*H*)-tetrone (**I**) (Florke, 2019 ▸) and 2-ethyl-12,12-di­meth­yl-4,6,7,8,9,9a-hexa­hydro-1*H*-4,9-[1,2]-epi­cyclo­buta­ben­zo[*f*]iso­indole-1,3,5(2*H*,3a*H*)trione (**II**) (Ma & Gu, 2006 ▸). In these two structures, the fused-ring systems are not planar. Compound **I** crystallizes in the monoclinic crystal system, space group *P*2_1_. The five- and six-membered rings are *cis*-fused. The crystal structure is characterized by the presence of C—H⋯O hydrogen bonds. Compound **II** crystallizes in the ortho­rhom­bic crystal system, space group *Pbca*. The mol­ecules are linked by C—H⋯O hydrogen bonds, and the crystal packing also features C—H⋯π inter­actions.

## Synthesis and crystallization

*N*-Allyl-*N*-phenyl­acryl­amide (0.30 mmol), 3-iodo­cyclo­hex-2-en-1-one (0.36 mmol), PdCl_2_(PPh_3_)_2_ (5 mol%, 0.015 mmol, 10.5 mg), TCAB (3,4,3′.4′-tetrachloroazobenzene) (10 mol%, 0.03 mmol, 8.33 mg) and K_2_CO_3_ (0.36 mmol, 49.68 mg) were stirred in DMSO (5.0 mL) at 403 K in a 20 mL tube under an N_2_ atmosphere. When the reaction was complete (detected by TLC), the mixture was cooled to room temperature. The reaction was quenched with HCl (5%, 10 mL) and extracted with Et_2_O (3 × 10 mL). The combined organic layers were dried over anhydrous MgSO_4_ and then evaporated under vacuum. The residue was purified by column chromatography on silica gel using *n*-hexa­ne/ethyl acetate (10:1 *v*:*v*) as eluent to afford the compound as a white solid. Part of the purified product was redissolved in *n*-hexa­ne/ethyl acetate and colourless crystals suitable for X-ray diffraction were formed after slow evaporation for several days.

Spectroscopic data: IR (film) 2962, 2920, 2885, 1687, 1662, 1619, 1169, 757 cm^−1^; ^1^H NMR (500 MHz, CDCl_3_): δ = 7.64–7.62 (*m*, 2H), 7.41–7.37 (*m*, 2H), 7.18–7.15 (*m*, 1H), 3.96–3.93 (*m*, 1H), 3.68–3.64 (*m*, 1H), 2.92–2.90 (*m*, 1H), 2.64–2.61 (*m*, 1H), 2.43–2.27 (*m*, 6H), 2.12–2.09 (*m*, 2H), 1.10 (*s*, 3H), 1.03 (*s*, 3H) ppm; ^13^C NMR (125 MHz, CDCl3): δ = 198.8, 173.7, 153.8, 139.6, 130.7, 128.9, 124.4, 119.6, 53.0, 51.4, 45.7, 45.1, 36.6, 33.1, 32.3, 29.4, 27.1, 26.3 ppm.

## Refinement

Crystal data, data collection and structure refinement details are summarized in Table 2 ▸. All H atoms were positioned geometrically with C—H = 0.93–0.98 Å and refined as riding atoms. The constraint *U*_iso_(H) = 1.2*U*_eq_(C) or 1.5*U*_eq_(C-meth­yl) was applied in all case. Atoms C8 and C9 are disordered over two positions (*A* and *B*) in a 0.805 (10):0.195 (10) occupancy ratio.

## Supplementary Material

Crystal structure: contains datablock(s) I. DOI: 10.1107/S2056989022002353/jy2017sup1.cif

Structure factors: contains datablock(s) I. DOI: 10.1107/S2056989022002353/jy2017Isup2.hkl

Supporting information file. DOI: 10.1107/S2056989022002353/jy2017Isup3.cml

CCDC reference: 2155680

Additional supporting information:  crystallographic information; 3D view; checkCIF report

## Figures and Tables

**Table 1:** Hydrogen-bond geometry (Å, °) *Cg*3 is the centroid of the C1–C6 ring.

*D*—H⋯*A*	*D*—H	H⋯*A*	*D*⋯*A*	*D*—H⋯*A*
C2—H2⋯O1	0.93	2.24	2.864 (3)	124
C3—H3⋯O2^i^	0.93	2.53	3.4513 (3)	170
C11—H11*A*⋯*Cg*3^ii^	0.97	2.73	3.688 (3)	168
C11—H11*D*⋯*Cg*3^ii^	0.97	2.95	3.688 (3)	134
C14—H14*A*⋯*Cg*3^iii^	0.97	2.70	3.609 (3)	156
C14—H14*D*⋯*Cg*3^iii^	0.97	2.90	3.609 (3)	131

Symmetry codes: (i) 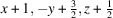 ; (ii) 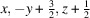 ; (iii) 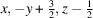 .

**Table 2:** Experimental details

Crystal data	
Chemical formula	C_20_H_23_NO_2_
*M*_r_	309.39
Crystal system, space group	Monoclinic, *P*2_1_/*c*
Temperature (K)	200
*a*, *b*, *c* (Å)	5.7062 (4), 34.009 (3), 8.5042 (8)
β (°)	98.178 (7)
*V* (Å^3^)	1633.5 (2)
*Z*	4
Radiation type	Mo *K*α
μ (mm^−1^)	0.08
Crystal size (mm)	0.12 × 0.1 × 0.08

Data collection	
Diffractometer	Rigaku Oxford Diffraction SuperNova, Dual, Cu at zero, AtlasS2
Absorption correction	Multi-scan (*CrysAlis PRO*; Rigaku OD, 2015 ▸)
*T*_min_, *T*_max_	0.621, 1.000
No. of measured, independent and observed [*I* > 2σ(*I*)] reflections	6736, 2874, 2046
*R*_int_	0.035
(sin θ/λ)_max_ (Å^−1^)	0.595

Refinement	
*R*[*F* ^2^ > 2σ(*F* ^2^)], *wR*(*F* ^2^), *S*	0.056, 0.143, 1.05
No. of reflections	2874
No. of parameters	229
H-atom treatment	H-atom parameters constrained
Δρ_max_, Δρ_min_ (e Å^−3^)	0.33, −0.31

Computer programs: *CrysAlis PRO* (Rigaku OD, 2015 ▸), *SHELXS* (Sheldrick, 2008 ▸), *SHELXL2017/1* (Sheldrick, 2015 ▸) and *OLEX2* (Dolomanov *et al.*, 2009 ▸).

## supplementary crystallographic information

### Crystal data

**Table:**

C_20_H_23_NO_2_	*F*(000) = 664
*M**_r_* = 309.39	*D*_x_ = 1.258 Mg m^−^^3^
Monoclinic, *P*2_1_/*c*	Mo *K*α radiation, λ = 0.71073 Å
*a* = 5.7062 (4) Å	Cell parameters from 2123 reflections
*b* = 34.009 (3) Å	θ = 2.4–27.9°
*c* = 8.5042 (8) Å	µ = 0.08 mm^−^^1^
β = 98.178 (7)°	*T* = 200 K
*V* = 1633.5 (2) Å^3^	Block, colourless
*Z* = 4	0.12 × 0.1 × 0.08 mm

### Data collection

**Table:**

Rigaku Oxford Diffraction SuperNova, Dual, Cu at zero, AtlasS2 diffractometer	2874 independent reflections
Radiation source: micro-focus sealed X-ray tube, SuperNova (Mo) X-ray Source	2046 reflections with *I* > 2σ(*I*)
Mirror monochromator	*R*_int_ = 0.035
Detector resolution: 10.5368 pixels mm^-1^	θ_max_ = 25.0°, θ_min_ = 2.4°
ω scans	*h* = −6→6
Absorption correction: multi-scan (CrysAlisPro; Rigaku OD, 2015)	*k* = −40→24
*T*_min_ = 0.621, *T*_max_ = 1.000	*l* = −10→8
6736 measured reflections	

### Refinement

**Table:**

Refinement on *F*^2^	Primary atom site location: structure-invariant direct methods
Least-squares matrix: full	Hydrogen site location: inferred from neighbouring sites
*R*[*F*^2^ > 2σ(*F*^2^)] = 0.056	H-atom parameters constrained
*wR*(*F*^2^) = 0.143	*w* = 1/[σ^2^(*F*_o_^2^) + (0.0507*P*)^2^ + 0.6477*P*] where *P* = (*F*_o_^2^ + 2*F*_c_^2^)/3
*S* = 1.05	(Δ/σ)_max_ < 0.001
2874 reflections	Δρ_max_ = 0.33 e Å^−^^3^
229 parameters	Δρ_min_ = −0.31 e Å^−^^3^
0 restraints	

### Special details

**Table:**

Geometry. All esds (except the esd in the dihedral angle between two l.s. planes) are estimated using the full covariance matrix. The cell esds are taken into account individually in the estimation of esds in distances, angles and torsion angles; correlations between esds in cell parameters are only used when they are defined by crystal symmetry. An approximate (isotropic) treatment of cell esds is used for estimating esds involving l.s. planes.
Refinement. Refinement of F^2^ against ALL reflections. The weighted R-factor wR and goodness of fit S are based on F^2^, conventional R-factors R are based on F, with F set to zero for negative F^2^. The threshold expression of F^2^ > 2sigma(F^2^) is used only for calculating R-factors(gt) etc. and is not relevant to the choice of reflections for refinement. R-factors based on F^2^ are statistically about twice as large as those based on F, and R- factors based on ALL data will be even larger. The C(8) and C(9) is disordered over two positions, site occupancies were refined. This refinement led to a 0.805 : 0.195 ratio in occupancy over two positions for C(8)and C(9).

### Fractional atomic coordinates and isotropic or equivalent isotropic displacement parameters (Å^2^)

**Table:**

	*x*	*y*	*z*	*U*_iso_*/*U*_eq_	Occ. (<1)
O1	1.0478 (3)	0.74574 (5)	0.8516 (3)	0.0677 (7)	
O2	0.3270 (3)	0.58244 (5)	0.4595 (2)	0.0497 (5)	
N1	0.6935 (3)	0.75875 (5)	0.6917 (2)	0.0319 (5)	
C1	0.6910 (4)	0.80046 (6)	0.6875 (3)	0.0312 (5)	
C2	0.8854 (4)	0.82281 (7)	0.7539 (3)	0.0369 (6)	
H2	1.021910	0.810308	0.802034	0.044*	
C3	0.8762 (4)	0.86346 (7)	0.7484 (3)	0.0414 (6)	
H3	1.006620	0.878040	0.793348	0.050*	
C4	0.6759 (4)	0.88257 (8)	0.6770 (3)	0.0444 (6)	
H4	0.670204	0.909893	0.674013	0.053*	
C5	0.4839 (4)	0.86066 (7)	0.6099 (3)	0.0427 (6)	
H5	0.348529	0.873425	0.561404	0.051*	
C6	0.4895 (4)	0.81998 (7)	0.6137 (3)	0.0377 (6)	
H6	0.359101	0.805634	0.567117	0.045*	
C7	0.5009 (4)	0.73534 (7)	0.6012 (3)	0.0359 (6)	
H7AA	0.506687	0.736741	0.487905	0.043*	0.805 (10)
H7AB	0.346778	0.744172	0.622299	0.043*	0.805 (10)
H7BC	0.454955	0.745403	0.494562	0.043*	0.195 (10)
H7BD	0.363048	0.733512	0.655672	0.043*	0.195 (10)
C8	0.5547 (7)	0.69357 (9)	0.6649 (5)	0.0308 (10)	0.805 (10)
H8	0.492969	0.690922	0.766149	0.037*	0.805 (10)
C9	0.8232 (7)	0.69415 (9)	0.6983 (5)	0.0314 (10)	0.805 (10)
H9	0.884344	0.692311	0.596540	0.038*	0.805 (10)
C10	0.8715 (4)	0.73524 (7)	0.7629 (3)	0.0436 (6)	
C11	0.9147 (4)	0.65947 (6)	0.7982 (3)	0.0346 (6)	
H11A	0.872712	0.662201	0.904263	0.042*	0.805 (10)
H11B	1.085889	0.658342	0.806781	0.042*	0.805 (10)
H11C	1.058398	0.665781	0.754796	0.042*	0.195 (10)
H11D	0.955968	0.655544	0.911742	0.042*	0.195 (10)
C12	0.8083 (4)	0.62222 (6)	0.7226 (3)	0.0314 (5)	
C13	0.6101 (4)	0.62233 (6)	0.6149 (3)	0.0320 (5)	
C14	0.4699 (4)	0.65896 (6)	0.5612 (3)	0.0336 (6)	
H14A	0.487383	0.664745	0.451778	0.040*	0.805 (10)
H14B	0.303290	0.654397	0.566237	0.040*	0.805 (10)
H14C	0.331267	0.660746	0.614939	0.040*	0.195 (10)
H14D	0.417279	0.657741	0.447692	0.040*	0.195 (10)
C15	0.9447 (4)	0.58530 (7)	0.7721 (3)	0.0384 (6)	
H15A	0.988461	0.585847	0.886542	0.046*	
H15B	1.089994	0.585636	0.725204	0.046*	
C16	0.8167 (4)	0.54667 (7)	0.7271 (3)	0.0410 (6)	
C17	0.6913 (4)	0.55090 (7)	0.5575 (3)	0.0454 (7)	
H17A	0.808661	0.554226	0.486470	0.055*	
H17B	0.603869	0.526973	0.527100	0.055*	
C18	0.5238 (4)	0.58520 (7)	0.5385 (3)	0.0370 (6)	
C19	0.6359 (4)	0.53812 (8)	0.8399 (4)	0.0534 (7)	
H19A	0.716904	0.534762	0.945931	0.080*	
H19B	0.550517	0.514530	0.806668	0.080*	
H19C	0.527086	0.559706	0.837605	0.080*	
C20	0.9935 (4)	0.51259 (7)	0.7373 (4)	0.0546 (8)	
H20A	1.107312	0.517452	0.666574	0.082*	
H20B	0.910590	0.488566	0.707522	0.082*	
H20C	1.073535	0.510389	0.844127	0.082*	
C21	0.733 (3)	0.6949 (3)	0.766 (2)	0.029 (4)	0.195 (10)
H21	0.613612	0.695152	0.838586	0.035*	0.195 (10)
C22	0.633 (3)	0.6964 (4)	0.602 (2)	0.032 (4)	0.195 (10)
H22	0.757910	0.697703	0.533654	0.039*	0.195 (10)

### Atomic displacement parameters (Å^2^)

**Table:**

	*U*^11^	*U*^22^	*U*^33^	*U*^12^	*U*^13^	*U*^23^
O1	0.0528 (11)	0.0479 (12)	0.0885 (16)	−0.0036 (9)	−0.0382 (11)	−0.0010 (11)
O2	0.0419 (10)	0.0464 (11)	0.0556 (12)	−0.0054 (8)	−0.0106 (9)	−0.0023 (9)
N1	0.0279 (10)	0.0344 (11)	0.0323 (11)	−0.0017 (8)	0.0003 (8)	−0.0006 (9)
C1	0.0305 (12)	0.0376 (13)	0.0257 (12)	−0.0018 (10)	0.0050 (9)	−0.0018 (10)
C2	0.0308 (12)	0.0440 (15)	0.0351 (14)	−0.0022 (10)	0.0022 (10)	0.0006 (11)
C3	0.0404 (14)	0.0455 (15)	0.0379 (15)	−0.0094 (11)	0.0039 (11)	−0.0036 (12)
C4	0.0539 (16)	0.0395 (14)	0.0394 (15)	−0.0004 (12)	0.0056 (12)	−0.0007 (12)
C5	0.0424 (14)	0.0428 (15)	0.0419 (15)	0.0069 (11)	0.0020 (12)	0.0019 (12)
C6	0.0333 (12)	0.0429 (14)	0.0358 (14)	−0.0011 (10)	0.0012 (10)	−0.0022 (11)
C7	0.0264 (11)	0.0390 (14)	0.0394 (15)	−0.0007 (10)	−0.0051 (10)	−0.0027 (11)
C8	0.022 (2)	0.0399 (18)	0.030 (2)	−0.0030 (13)	0.0017 (16)	0.0019 (15)
C9	0.022 (2)	0.0405 (18)	0.031 (2)	0.0015 (13)	0.0032 (16)	−0.0008 (14)
C10	0.0362 (13)	0.0404 (14)	0.0488 (17)	−0.0015 (11)	−0.0122 (12)	0.0034 (12)
C11	0.0277 (11)	0.0396 (14)	0.0348 (14)	0.0007 (9)	−0.0013 (10)	0.0007 (11)
C12	0.0259 (11)	0.0365 (13)	0.0327 (13)	0.0002 (9)	0.0072 (10)	0.0003 (10)
C13	0.0275 (11)	0.0371 (13)	0.0318 (13)	0.0006 (9)	0.0058 (10)	−0.0008 (10)
C14	0.0274 (11)	0.0391 (14)	0.0327 (14)	−0.0018 (9)	−0.0011 (10)	−0.0007 (11)
C15	0.0301 (12)	0.0413 (14)	0.0426 (15)	0.0033 (10)	0.0008 (10)	0.0016 (12)
C16	0.0328 (13)	0.0385 (14)	0.0507 (17)	0.0037 (10)	0.0028 (11)	0.0028 (12)
C17	0.0430 (14)	0.0365 (14)	0.0545 (18)	0.0007 (11)	−0.0010 (12)	−0.0057 (13)
C18	0.0356 (13)	0.0407 (14)	0.0340 (14)	−0.0042 (10)	0.0020 (11)	0.0029 (11)
C19	0.0433 (15)	0.0527 (17)	0.064 (2)	0.0019 (12)	0.0058 (13)	0.0148 (14)
C20	0.0474 (15)	0.0400 (15)	0.074 (2)	0.0076 (12)	−0.0004 (14)	−0.0008 (14)
C21	0.025 (8)	0.035 (7)	0.031 (9)	0.009 (5)	0.016 (7)	0.005 (6)
C22	0.034 (9)	0.028 (7)	0.041 (10)	−0.008 (5)	0.027 (7)	−0.001 (6)

### Geometric parameters (Å, º)

**Table:**

O1—C10	1.222 (3)	C11—H11C	0.9700
O2—C18	1.228 (3)	C11—H11D	0.9700
N1—C1	1.419 (3)	C11—C12	1.509 (3)
N1—C7	1.481 (3)	C11—C21	1.587 (13)
N1—C10	1.365 (3)	C12—C13	1.350 (3)
C1—C2	1.396 (3)	C12—C15	1.505 (3)
C1—C6	1.397 (3)	C13—C14	1.515 (3)
C2—H2	0.9300	C13—C18	1.472 (3)
C2—C3	1.384 (3)	C14—H14A	0.9700
C3—H3	0.9300	C14—H14B	0.9700
C3—C4	1.379 (3)	C14—H14C	0.9700
C4—H4	0.9300	C14—H14D	0.9700
C4—C5	1.379 (3)	C14—C22	1.588 (14)
C5—H5	0.9300	C15—H15A	0.9700
C5—C6	1.384 (3)	C15—H15B	0.9700
C6—H6	0.9300	C15—C16	1.525 (3)
C7—H7AA	0.9700	C16—C17	1.523 (4)
C7—H7AB	0.9700	C16—C19	1.533 (3)
C7—H7BC	0.9700	C16—C20	1.531 (3)
C7—H7BD	0.9700	C17—H17A	0.9700
C7—C8	1.535 (4)	C17—H17B	0.9700
C7—C22	1.524 (14)	C17—C18	1.502 (3)
C8—H8	0.9800	C19—H19A	0.9600
C8—C9	1.518 (7)	C19—H19B	0.9600
C8—C14	1.509 (4)	C19—H19C	0.9600
C9—H9	0.9800	C20—H20A	0.9600
C9—C10	1.513 (4)	C20—H20B	0.9600
C9—C11	1.503 (4)	C20—H20C	0.9600
C10—C21	1.586 (14)	C21—H21	0.9800
C11—H11A	0.9700	C21—C22	1.43 (3)
C11—H11B	0.9700	C22—H22	0.9800
			
C1—N1—C7	121.36 (17)	C13—C12—C15	122.8 (2)
C10—N1—C1	126.93 (19)	C15—C12—C11	114.87 (19)
C10—N1—C7	111.47 (18)	C12—C13—C14	124.2 (2)
C2—C1—N1	122.0 (2)	C12—C13—C18	119.5 (2)
C2—C1—C6	118.6 (2)	C18—C13—C14	116.28 (19)
C6—C1—N1	119.41 (19)	C8—C14—C13	110.7 (2)
C1—C2—H2	119.8	C8—C14—H14A	109.5
C3—C2—C1	120.4 (2)	C8—C14—H14B	109.5
C3—C2—H2	119.8	C13—C14—H14A	109.5
C2—C3—H3	119.6	C13—C14—H14B	109.5
C4—C3—C2	120.7 (2)	C13—C14—H14C	109.9
C4—C3—H3	119.6	C13—C14—H14D	109.9
C3—C4—H4	120.4	C13—C14—C22	108.9 (6)
C3—C4—C5	119.2 (2)	H14A—C14—H14B	108.1
C5—C4—H4	120.4	H14C—C14—H14D	108.3
C4—C5—H5	119.5	C22—C14—H14C	109.9
C4—C5—C6	121.1 (2)	C22—C14—H14D	109.9
C6—C5—H5	119.5	C12—C15—H15A	108.3
C1—C6—H6	120.0	C12—C15—H15B	108.3
C5—C6—C1	120.0 (2)	C12—C15—C16	116.00 (19)
C5—C6—H6	120.0	H15A—C15—H15B	107.4
N1—C7—H7AA	111.3	C16—C15—H15A	108.3
N1—C7—H7AB	111.3	C16—C15—H15B	108.3
N1—C7—H7BC	112.2	C15—C16—C19	110.3 (2)
N1—C7—H7BD	112.2	C15—C16—C20	110.48 (19)
N1—C7—C8	102.57 (19)	C17—C16—C15	107.5 (2)
N1—C7—C22	97.7 (6)	C17—C16—C19	110.1 (2)
H7AA—C7—H7AB	109.2	C17—C16—C20	110.0 (2)
H7BC—C7—H7BD	109.8	C20—C16—C19	108.5 (2)
C8—C7—H7AA	111.3	C16—C17—H17A	109.1
C8—C7—H7AB	111.3	C16—C17—H17B	109.1
C22—C7—H7BC	112.2	H17A—C17—H17B	107.9
C22—C7—H7BD	112.2	C18—C17—C16	112.4 (2)
C7—C8—H8	108.4	C18—C17—H17A	109.1
C9—C8—C7	101.5 (3)	C18—C17—H17B	109.1
C9—C8—H8	108.4	O2—C18—C13	122.0 (2)
C14—C8—C7	119.0 (3)	O2—C18—C17	121.2 (2)
C14—C8—H8	108.4	C13—C18—C17	116.8 (2)
C14—C8—C9	110.5 (4)	C16—C19—H19A	109.5
C8—C9—H9	108.1	C16—C19—H19B	109.5
C10—C9—C8	101.9 (3)	C16—C19—H19C	109.5
C10—C9—H9	108.1	H19A—C19—H19B	109.5
C11—C9—C8	110.8 (4)	H19A—C19—H19C	109.5
C11—C9—H9	108.1	H19B—C19—H19C	109.5
C11—C9—C10	119.3 (3)	C16—C20—H20A	109.5
O1—C10—N1	126.8 (2)	C16—C20—H20B	109.5
O1—C10—C9	125.9 (2)	C16—C20—H20C	109.5
O1—C10—C21	127.6 (5)	H20A—C20—H20B	109.5
N1—C10—C9	107.1 (2)	H20A—C20—H20C	109.5
N1—C10—C21	100.0 (5)	H20B—C20—H20C	109.5
C9—C11—H11A	109.8	C10—C21—H21	113.3
C9—C11—H11B	109.8	C11—C21—C10	110.2 (10)
C9—C11—C12	109.4 (2)	C11—C21—H21	113.3
H11A—C11—H11B	108.2	C22—C21—C10	94.6 (12)
H11C—C11—H11D	108.1	C22—C21—C11	110.6 (14)
C12—C11—H11A	109.8	C22—C21—H21	113.3
C12—C11—H11B	109.8	C7—C22—C14	114.8 (10)
C12—C11—H11C	109.5	C7—C22—H22	111.1
C12—C11—H11D	109.5	C14—C22—H22	111.1
C12—C11—C21	110.6 (5)	C21—C22—C7	99.4 (13)
C21—C11—H11C	109.5	C21—C22—C14	108.8 (14)
C21—C11—H11D	109.5	C21—C22—H22	111.1
C13—C12—C11	122.3 (2)		
			
O1—C10—C21—C11	−41.8 (15)	C10—N1—C7—C22	−13.4 (8)
O1—C10—C21—C22	−155.8 (9)	C10—C9—C11—C12	169.8 (3)
N1—C1—C2—C3	−179.6 (2)	C10—C21—C22—C7	−58.3 (14)
N1—C1—C6—C5	179.5 (2)	C10—C21—C22—C14	−178.7 (7)
N1—C7—C8—C9	−33.0 (4)	C11—C9—C10—O1	32.6 (6)
N1—C7—C8—C14	−154.5 (3)	C11—C9—C10—N1	−153.1 (3)
N1—C7—C22—C14	162.6 (11)	C11—C12—C13—C14	−1.6 (3)
N1—C7—C22—C21	46.7 (14)	C11—C12—C13—C18	176.29 (19)
N1—C10—C21—C11	163.7 (8)	C11—C12—C15—C16	167.2 (2)
N1—C10—C21—C22	49.7 (14)	C11—C21—C22—C7	−171.9 (7)
C1—N1—C7—C8	−170.1 (3)	C11—C21—C22—C14	67.7 (19)
C1—N1—C7—C22	161.4 (8)	C12—C11—C21—C10	−151.1 (7)
C1—N1—C10—O1	9.5 (4)	C12—C11—C21—C22	−47.8 (17)
C1—N1—C10—C9	−164.7 (3)	C12—C13—C14—C8	−10.3 (4)
C1—N1—C10—C21	164.3 (7)	C12—C13—C14—C22	19.4 (8)
C1—C2—C3—C4	−0.3 (3)	C12—C13—C18—O2	168.4 (2)
C2—C1—C6—C5	−1.2 (3)	C12—C13—C18—C17	−13.5 (3)
C2—C3—C4—C5	−0.3 (4)	C12—C15—C16—C17	42.8 (3)
C3—C4—C5—C6	0.2 (4)	C12—C15—C16—C19	−77.2 (3)
C4—C5—C6—C1	0.6 (3)	C12—C15—C16—C20	162.8 (2)
C6—C1—C2—C3	1.0 (3)	C13—C12—C15—C16	−14.9 (3)
C7—N1—C1—C2	−171.0 (2)	C13—C14—C22—C7	−162.6 (9)
C7—N1—C1—C6	8.4 (3)	C13—C14—C22—C21	−52.3 (17)
C7—N1—C10—O1	−176.1 (3)	C14—C8—C9—C10	165.6 (2)
C7—N1—C10—C9	9.7 (3)	C14—C8—C9—C11	−66.4 (5)
C7—N1—C10—C21	−21.3 (7)	C14—C13—C18—O2	−13.5 (3)
C7—C8—C9—C10	38.4 (5)	C14—C13—C18—C17	164.6 (2)
C7—C8—C9—C11	166.4 (2)	C15—C12—C13—C14	−179.4 (2)
C7—C8—C14—C13	159.4 (3)	C15—C12—C13—C18	−1.4 (3)
C8—C9—C10—O1	155.0 (3)	C15—C16—C17—C18	−56.9 (2)
C8—C9—C10—N1	−30.7 (4)	C16—C17—C18—O2	−137.6 (2)
C8—C9—C11—C12	52.0 (5)	C16—C17—C18—C13	44.2 (3)
C9—C8—C14—C13	42.5 (5)	C18—C13—C14—C8	171.7 (3)
C9—C11—C12—C13	−19.3 (4)	C18—C13—C14—C22	−158.6 (8)
C9—C11—C12—C15	158.6 (3)	C19—C16—C17—C18	63.3 (3)
C10—N1—C1—C2	2.9 (3)	C20—C16—C17—C18	−177.2 (2)
C10—N1—C1—C6	−177.7 (2)	C21—C11—C12—C13	13.7 (8)
C10—N1—C7—C8	15.1 (3)	C21—C11—C12—C15	−168.4 (8)

### Hydrogen-bond geometry (Å, º)

*Cg*3 is the centroid of the C1–C6 ring.

**Table:**

*D*—H···*A*	*D*—H	H···*A*	*D*···*A*	*D*—H···*A*
C2—H2···O1	0.93	2.24	2.864 (3)	124
C3—H3···O2^i^	0.93	2.53	3.4513 (3)	170
C11—H11*A*···*Cg*3^ii^	0.97	2.73	3.688 (3)	168
C11—H11*D*···*Cg*3^ii^	0.97	2.95	3.688 (3)	134
C14—H14*A*···*Cg*3^iii^	0.97	2.70	3.609 (3)	156
C14—H14*D*···*Cg*3^iii^	0.97	2.90	3.609 (3)	131

Symmetry codes: (i) *x*+1, −*y*+3/2, *z*+1/2; (ii) *x*, −*y*+3/2, *z*+1/2; (iii) *x*, −*y*+3/2, *z*−1/2.
